# A Comparison of Hip Spica Casting to Short Leg Casts and Bar after Hip Reconstruction in Cerebral Palsy

**DOI:** 10.7759/cureus.8028

**Published:** 2020-05-08

**Authors:** Uyen Truong, Tonye Sylvanus, Trever M Koester, Chantel C Barney, Andrew G Georgiadis, Jennifer Carpenter, Walter Truong, Susan A Novotny

**Affiliations:** 1 Orthopaedic Surgery, Gillette Children's Specialty Healthcare, Saint Paul, USA; 2 Pain and Comfort Research, Gillette Children's Specialty Healthcare, Saint Paul, USA; 3 Department of Educational Psychology, University of Minnesota, Minneapolis, USA; 4 Research Administration, Gillette Children's Specialty Healthcare, Saint Paul, USA; 5 Rehabilitation Science Graduate Program, University of Minnesota, Minneapolis, USA

**Keywords:** cerebral palsy, neuromuscular hip subluxation, spastic hip subluxation, spica cast

## Abstract

Background

Immobilization after hip reconstruction in children with cerebral palsy varies according to surgeon preference. The effect of postoperative immobilization on postoperative pain is unknown. Success in achieving hip stability and complications may also differ depending on the immobilization technique utilized.

Questions/purposes

Using retrospective data, we aimed to evaluate: (a) what effect does postoperative immobilization with hip spica casting versus short leg casts and bar (SLCaB); have on pain and pain management in children with quadriplegic cerebral palsy undergoing femoral and/or pelvic osteotomy? and (b) Do complications and radiographic outcomes differ between those treated postoperatively with hip spica casting and those in short leg casts?

Materials and Methods

Children with quadriplegic cerebral palsy (GMFCS IV-V, mean age 7.8 years [range: 3-15 years]) undergoing femoral or pelvic osteotomy between 2012 and 2014 in the treatment of spastic hip subluxation were reviewed. Modes of immobilization were compared, between spica casting (n=15) and SLCaB (n=12). Preoperative, perioperative, and postoperative pain was quantified between groups. In-hospital epidural dosage, morphine equivalent dosages (MED), adjunctive medications, early maintenance of radiographic hip stability, and all complications were noted and analyzed.

Results

Children were more likely to have spica cast immobilization if they were younger. Postoperative pain scores were similar between groups, with comparable patterns of epidural and MED administered during hospitalization. Spica casts were often flared up during hospitalization, but skin ulcers were uncommon and comparable between the two groups. Within 12 months of surgery, more ipsilateral femur fractures were observed distant to implants in the hip spica group, although the incidence of fractures did not meet statistical thresholds.

Conclusion

Spica casting and SLCaB after neuromuscular hip reconstruction did not show a difference in hip stability, narcotic pain medication usage or complication profile.

## Introduction

The ideal post-surgical immobilization after femoral and pelvic osteotomy for children with cerebral palsy is debated, and practice varies widely. Current literature suggests hip spica casting is the most common method of immobilization for this population [1-10]. Theoretic advantages of hip spica casting include protection of the surgical wound, reduced motion at the osteotomy site(s), mitigating hip flexion contractures, protection of osteotomies in patients with relative osteopenia, and possibly reducing post-operative pain. Complications of hip spica casting include skin sores, disuse-mediated osteopenia, joint stiffness, and increased caregiver burden for care during the casting period [1-4, 11-17]. Short leg casts and bar (SLCaB), abduction braces, and wedges have consequently emerged and may be equally effective following hip reconstruction [18-19]. Such fully or partially removable immobilization may allow easier hygiene or permit the patient to sit and initiate rehabilitation therapy within days of surgery [20]. Concerns with SLCaB, and other less-rigid forms of immobilization, include increased pain, risk of fixation failure, wound breakdown, and loss of reduction due to insufficient immobilization [20].

In one retrospective investigation of hip reconstruction in cerebral palsy, complication rates for spica immobilization versus no casting were reported to be equivalent [21]. Other investigations in cerebral palsy have found spica casting to be a risk factor for skin complications or osteoporotic fractures [11, 13]. It is unknown if spica casting in this setting has any effect on pain medication administration.

The primary aim of this study was to compare pain and pain management in children with quadriplegic cerebral palsy undergoing femoral and/or pelvic osteotomy immobilized in either a hip spica cast or SLCaB. We hypothesized that those placed in hip spica casts would have lower pain scores and a reduced need for opioid medications compared to those placed in SLCaB forms of immobilization. The secondary aim of this study was to determine complications and radiographic outcomes in both groups.

## Materials and methods

Study design and inclusion criteria

A retrospective cohort study was conducted to compare the effect of hip spica casting versus SLCaB on pain levels following proximal femoral and/or pelvic osteotomy surgery in the setting of neuromuscular hip subluxation. Individuals between the ages of three and 17 were included in the study if they had a diagnosis of quadriplegic cerebral palsy functioning at a Gross Motor Function Classification System (GMFCS) level IV or V, had neuromuscular hip dysplasia, underwent a femoral and/or pelvic osteotomy between 2012 and 2014, were immobilized postoperatively in either a spica cast or in SLCaB, and had a minimum of one-year follow-up. Individuals were excluded from the study if they had diagnoses other than cerebral palsy, GMFCS levels I-III, previous hip surgery, immobilization in a brace or pillow, a patient-controlled analgesia pump for pain control, or an epidural dosage differing from standard of care. All data were collected from a single tertiary care center, with inclusion of patients from one of nine pediatric orthopaedic surgeons.

Compliance with ethical standards

All procedures performed in this study involving human participants were in accordance with the ethical standards of the institutional and/or national research committee and with the 1964 Helsinki declaration and its later amendments or comparable ethical standards as well as the relevant regulations of the US Health Insurance Portability and Accountability Act. This study was approved by the Institutional Review Board of our local university and all data was collected at a single center. Informed consent was not required by our Institutional Review Board.

Demographics and surgery

Demographic information collected included: age at surgery, gender, functional status, and underlying diagnosis. All femoral osteotomies were performed by a standardized technique and implant type: subperiosteal exposure, intertrochanteric osteotomy, and fixation with 90° or 100° blade plates utilizing three distal (subtrochanteric), bicortical cortex screws. The decision for use of spica casting or SLCaB was based on surgeon preference. Unilateral and bilateral procedures were included. Surgical and inpatient data included location and type of osteotomy, surgery duration, duration of inpatient stay, complications (classified according to Clavien-Dindo), and medications and dosing for the inpatient stay and discharge (described below) [22].

Pain scores and medications

Pre-surgical pain level was defined as the most recent pain score within a year prior to surgery. Peri-operative pain scores were extracted in hourly intervals throughout the length of stay; the maximum daily pain score was used for analyses. Pre-operative pain scores were available for 22 children and peri-operative pain scores were available for all 27 children. Pain assessment scales routinely used in the clinic included the Faces Pain scale; Face, Legs, Activity, Cry, Consolability (FLACC), a numeric rating scale, or an observational scale [23-24]. All of these pain scales result in a pain score ranging from zero to 10. Thus, all scores were collapsed into a single zero to 10 score. Of note, scoring properties of these scales differ. Specifically, the FLACC requires each of five domains (e.g., face, legs, activity, etc.) to be scored zero to two for intensity. The verbal report of pain using the numeric rating scale was scored on an 11-point scale with zero meaning “no pain,” and 10 meaning “the worst pain imaginable.” The observational pain scale is scored on an 11-point scale with zero meaning “no indicators of pain” were observed, and 10 meaning the “worst indicators of pain” were observed.

Standard inpatient anesthesia consisted of an indwelling epidural catheter infusion of 0.1% ropivacaine with 1 mg/ml clonidine infused at patient-specific rates. Weight-adjusted epidural load (ml/kg) was calculated on an individual basis considering the rate of infusion, duration of use, bolus doses, and body weight. All medications that would potentially provide an analgesic effect administered between the time of surgery and discharge were recorded. Oral and intravenous pain medications were documented on an hourly basis in milligrams. Opioids were converted to morphine equivalent dosing (MED) using standard conversion tables, and a weight-adjusted daily total (MED/kg/day) was calculated for each postoperative day [25-26]. Standard adjunctive inpatient medications included: acetaminophen, diazepam, hydroxyzine, and oxycodone, and these were continued as outpatient prescriptions.

Postoperative clinic visits

Postoperative pain was assessed at the first (approximately one month) and second (approximately three months) clinic visits in two ways: first, pain that was experienced since the last visit (i.e., since discharge or one-month visit) and secondly, current pain levels on the day of the clinic visit. Postoperative pain data were available for 26 children at the first clinic visit and 25 at the second clinic visit. Secondary outcomes for clinic visits included: immobilization status, weight-bearing status, duration of immobilization, and complications (immobilization-related or general). Fractures that occurred within 12 months of surgery were also noted, along with time to final follow-up.

Orthopaedic outcomes

The orthopaedic-related outcomes of hip reconstruction were extracted from radiographs and medical records for all 27 children. Hip subluxation was assessed on anterior-posterior radiographs preoperatively and at three months post-surgery by Reimer’s Migration Percentage (MP), and dysplasia was assessed by the acetabular index [27-28]. All complications were recorded.

Statistical analyses

Demographic data, surgical details, postoperative treatment protocols, radiographic measurements, fracture incidence, and complications were compared between the spica and SLCaB groups using two-sided t-tests or Mann-Whitney Rank Sum tests for continuous variables, and chi-square tests or Fisher exact tests for categorical variables. The incidence of additional surgeries and fractures were reported by individual children and the percentage of the group.

Maximum daily inpatient pain scores (days zero to six), morphine (days zero to six, MED/kg), epidural loads (days zero to three, ml/kg), and other medications (days zero to six, mg/kg) were analyzed using two-way repeated measures ANOVA to determine the effect of treatment (spica versus SLCaB) and time (day of inpatient stay) as the repeated factor. Holmes-Sidak post hoc tests were performed when significant interactions were detected.

A combined total sum of maximum pain scores and medication intake were calculated on an individual patient basis, and values were compared between groups using two-sided t-tests or Mann-Whitney Rank Sum tests. SigmaPlot (Systat Software Inc., San Jose, California) was used for all statistical analyses, with significance set at P<0.05.

## Results

Demographics and surgery

**Figure 1:**
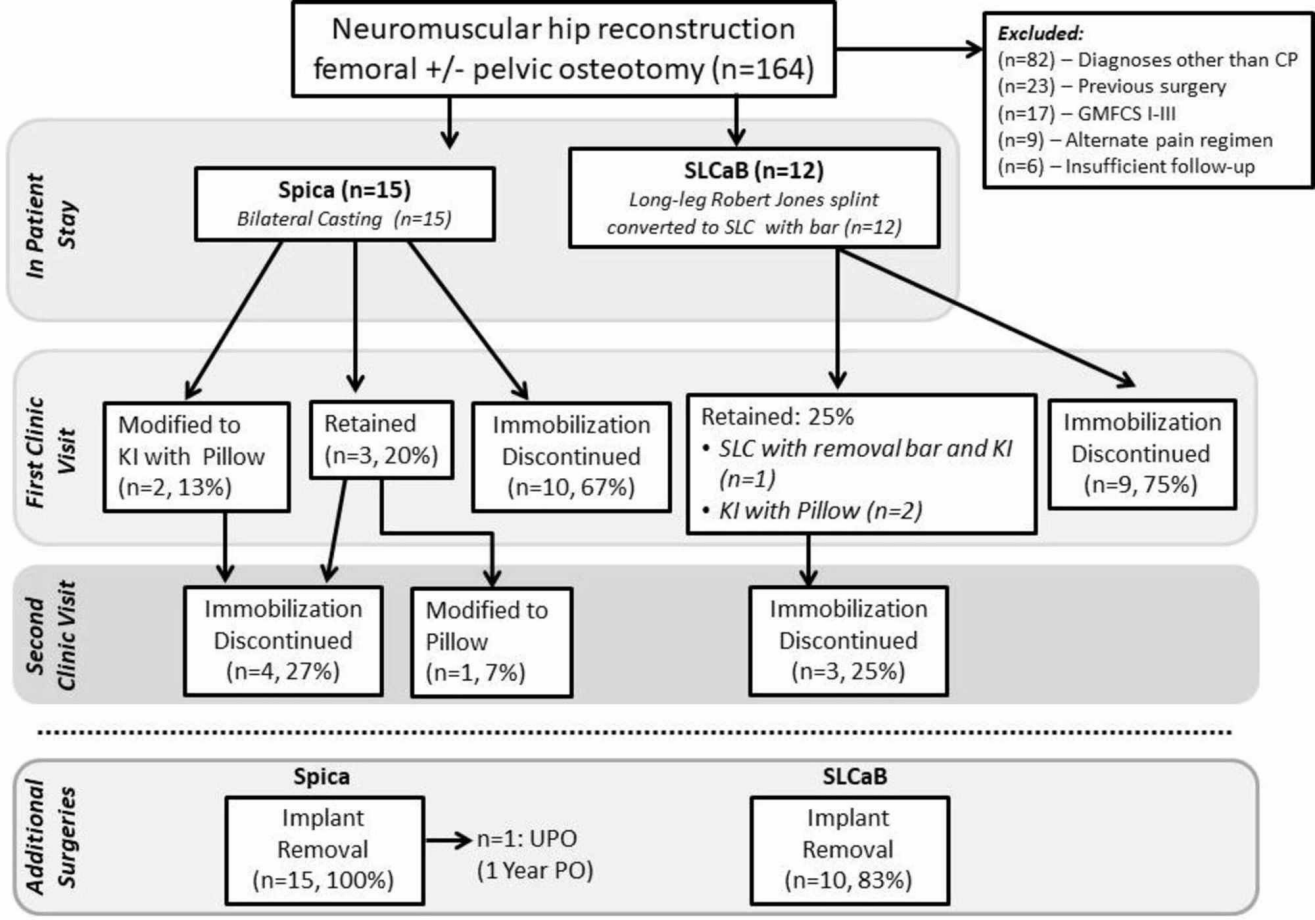
Treatment pathways for immobilization Cerebral palsy (CP), Gross Motor Function Classification System (GMFCS), short leg casts and bar (SLCaB), short leg cast (SLC), knee immobilizer (KI), abduction pillow (Pillow), abduction brace (Brace), postoperative (PO), unilateral pelvic osteotomy (UPO).

One hundred and sixty-four children had femoral or pelvic osteotomies in 2012-2014, and 27 individuals met inclusion and exclusion criteria (spica n=15, SLCaB n=12, Figure 1). There was a significant difference between groups for age at surgery (P=0.038), while sex, height, and weight were equivalent between groups (Table 1). There were no differences in the proportion of femoral and femoral/pelvic osteotomies comprising the spica versus SLCaB group (P=0.830, Table 1). All reconstructions were bilateral, with the exception of one child in the SLCaB group. One open hip reduction was performed in a single child from the spica group. Post-surgical pathways for immobilization during pain assessment are represented in Figure 1. Spica casts were applied for 41 ± 9 days (range 23-103 days) and SLCaB were applied for 34 ± 9 days (range 23-47 days, P=0.1710). There was also no difference between the duration of surgery (P=0.393), duration of inpatient stay (P=0.606) or time to final follow-up between groups (P=0.751; Table 1).

**Table 1:** Demographics and surgical details The data in the above table represents number (%); mean ± standard deviation (range).

Variable	Spica (n=15)	SLCaB (n=12)	P-value
Sex			0.077
Female	10 (66.7%)	3 (25%)	
Male	5 (33.3%)	9 (75%)	
Age at surgery (y)	6.8 ± 2.2 (3.0 – 9.8)	9.1 ± 3.2 (5.1 – 15.1)	0.038
Height (cm)	108 ± 16 (80 – 127)	119 ± 11 (98 – 133)	0.102
Weight (kg)	19.2 ± 7.8 (9.3 – 38.5)	22.9 ± 6.3 (11.7 – 37.4)	0.194
Location of osteotomy			
Femoral	7 (47%)	6 (50%)	0.830
Pelvic and femoral	8 (53%)	6 (50%)	
Duration of surgery (hours)	7.1 ± 0.9 (5.7 – 8.5)	6.8 ± 1.2 (5.9 – 9.7)	0.393
Duration of inpatient stay (days)	4.9 ± 1.3 (3.0 – 8.0)	5.0 ± 0.9 (4.0 – 7.0)	0.606
Time to final follow-up visit (y)	3.3 ± 1.3 (0.9 – 4.8)	3.4 ± 1.5 (0.1 – 5.0)	0.751

Preoperative and perioperative pain scores

**Figure 2:**
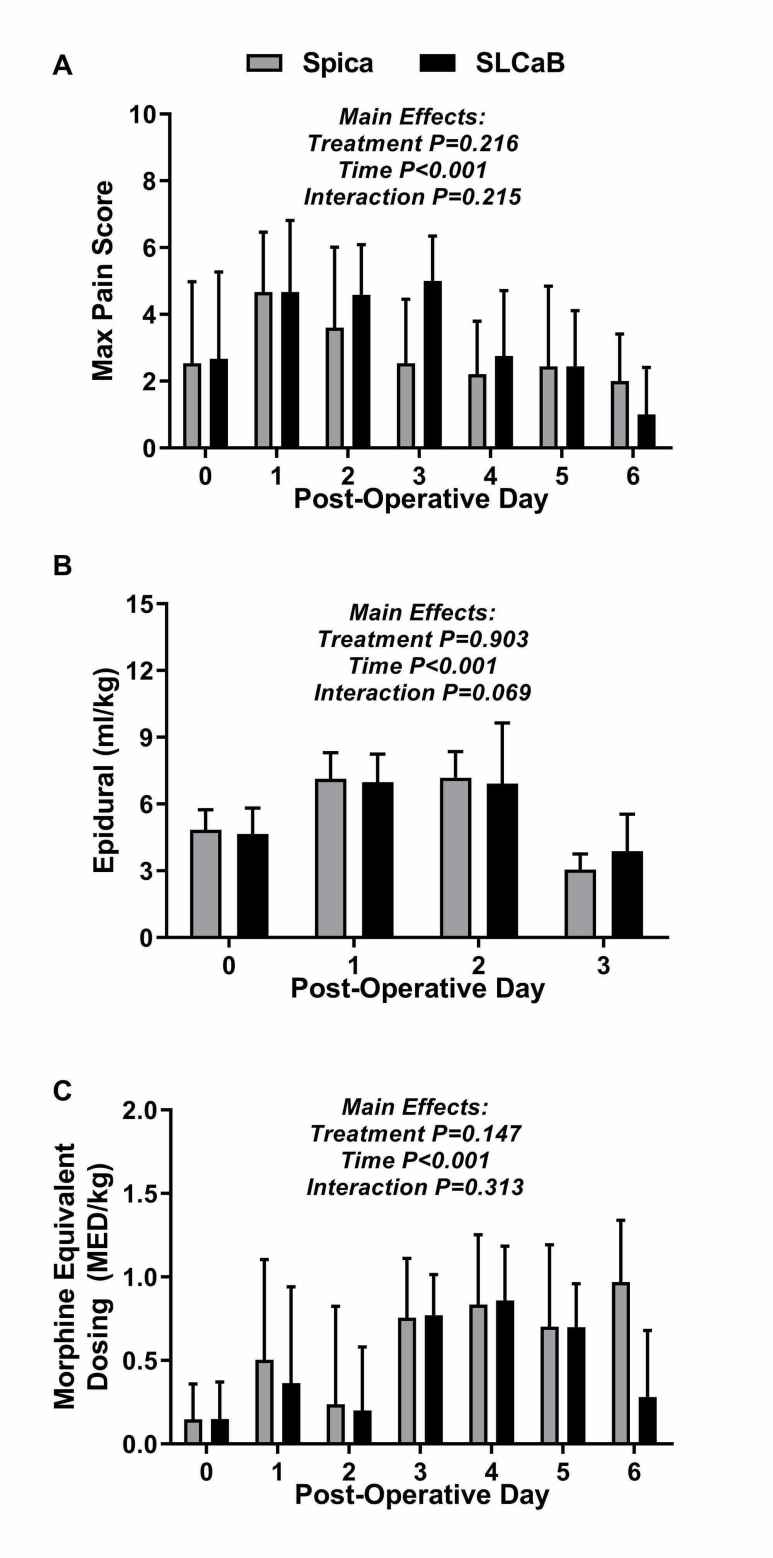
Pain scores, morphine equivalent dosing, and epidurals during inpatient stay The data in the above table represents mean and standard deviation. The main effects of the two-way analysis of variance (ANOVA) are listed above the bars. Holmes-Sidak post hoc test results: A) Main effect of time on Maximum Pain Score: Postoperative day one versus zero, one versus four, one versus five (P<0.001 for all). Postoperative day one versus six (P=0.004), two versus four (P=0.031), two versus five (P=0.034), two versus six (P=0.034). B) Epidural interactions: Within-group differences were the same for both spica and short leg casts and bar (SLCaB) and include postoperative day zero versus one, zero versus two, zero versus three, one versus three and two versus three (P<0.01 for all). C) Main effect of time on morphine equivalent dosing: Postoperative day zero versus three (P<0.001), zero versus four (P<0.001), zero versus five (P<0.001), one versus three (P=0.018), one versus four (P<0.001), two versus three (P<0.001), two versus four (P<0.001), two versus five (P=0.003). These results suggest no difference in overall pain scores between treatment groups, and no difference in epidural dosage or morphine equivalent dosages (MED) between spica or SLCaB groups. Epidural dosage peaked on days one and two in both groups, and MED peaked during days three to five in both groups.

At the preoperative clinic visit, only one of 22 patients (0/12 [0%] patients in the spica group and 1/10 [10%] patients in the SLCaB group, P=0.455) reported pain. Maximum pain scores during any specific inpatient day were not affected by the type of immobilization treatment (P= 0.216, Figure 2A). Maximum pain scores during the inpatient stay peaked on postoperative days one through three and decreased thereafter (Figure 2A) for both groups.

Peri-operative analgesia

Epidural analgesia was utilized from the day of surgery until postoperative day three in all children, save one individual in the SLCaB group who had epidural removal on day four. There was no main effect of treatment on epidural analgesia; however, a significant main effect of time was identified. Specifically, epidural load peaking on days one and two for both groups (Figure 2B).

There was no main effect of immobilization type for daily MED, however, there was a main effect of time (Figure 2C). Specifically, daily MED significantly increased at post-surgical days three through five (P<0.001), regardless of immobilization type.

Total weight- and length of stay-adjusted daily intake of acetaminophen, diazepam, ketorolac, and hydroxyzine during the inpatient stay did not differ between groups (P≥0.616; Table 2), and codeine, fentanyl, and tramadol were not used by any child during the inpatient stay.

**Table 2:** Group comparison of total medications given Data are reported in units of mg/kg and represent mean ± standard deviation (range).

Drug	Spica (n=15)	SLCaB (n=12)	P-value
Morphine equivalent	3.17 ± 1.77 (0.77 – 6.03)	2.92 ± 1.63 (1.06 – 6.61)	0.705
Acetaminophen	257.68 ± 98.17 (119.68 – 444.34)	239.85 ± 82.69 (80.25 – 413.64)	0.616
Diazepam	2.52 ± 1.83 (0.16 – 5.40)	2.56 ± 0.88 (0.99 – 3.70)	0.751
Hydroxyzine	4.53 ± 2.52 (0.46 – 8.93)	4.94 ± 2.15 (0.41 – 7.27)	0.655
Ketoralac	3.90 ± 2.87 (1.84 – 7.18)	4.12 ± 0.94 (3.59 – 5.79)	0.874

Postoperative outpatient pain

The occurrence of pain between the time of discharge and the first clinic visit was similar between groups (P=1.000, Table 3). At the first clinic visit, the proportion of individuals currently experiencing pain also did not differ by group, with 33% and 27% of individuals reporting pain in the spica and SLCaB groups, respectively (P=1.000, Table 3).

**Table 3:** Post-surgical pain In the above table, the data represent mean ± standard deviation (range), or number (%). *The fraction represents the incidence and number of available patients with data. Results may reflect pain immediately after immobilization was discontinued.

	Spica (n=15)	SLCaB (n=12)	P-value
First clinic visit			
Days since surgery	37 ± 20 (20 – 105)	34 ± 9 (23 – 47)	0.903
Pain experienced since discharge	2 (13%)	2 (17%)	1.000
Pain currently experienced	5/15* (33%)	3/11 (27%)	1.000
Second clinic visit			
Days since surgery	81 ± 58 (36 – 259)	84 ± 34 (41 – 141)	0.586
Pain experienced since first clinic visit	5 (33%)	3 (25%)	0.696
Pain currently experienced	7/15* (47%)	3/10 (30%)	0.678

Some individuals experienced pain between the first and second clinic visit in both groups (33% for spica and 25% for SLCaB, p=0.696, Table 3). The proportion of individuals experiencing pain at the second clinic visit (postoperative two- to three- months) was not different between groups (47% for spica vs 30% in SLCaB, Table 3).

Orthopaedic outcomes

Preoperative acetabular index measurements of the most dysplastic hip were equivalent between groups (SLCaB 29 ± 6 degrees (18-40 degrees) versus spica 33 ± 7 degrees (23-43 degrees), P=0.101). The pre-operative Reimer’s MP for the most subluxated hip was significantly different between groups, with the spica group having displaced hips (SLCaB 50 ± 12% (36-71%) versus spica 70 ± 20% (35-100%), P=0.010). Postoperative assessments of hip subluxation and dysplasia were performed on radiographs obtained a mean of 99 ± 57 days (range 36-261 days) after surgery. Four children in the spica group had an acetabular index >30 degrees compared to one child in the SLCaB group (P=0.342, Table 4), and one child in the spica group had a Reimer’s MP > 30% compared to zero children in the SLCaB group (P=1.000, Table 4).

**Table 4:** Postoperative orthopaedic outcomes In the above table, the number is represented (%). ^1^Fractures occurred non-adjacent to the surgical site at weeks 8, 18, 19, and 29 after surgery. ^2^Repeat osteotomy was a unilateral pelvic osteotomy conducted one year after surgery.

Outcome	Spica (n=15)	SLCaB (n=12)	P-value
Postoperative radiographic outcomes			
Acetabular angle >30 degrees	4 (27%)	1 (8%)	0.342
Reimer’s migration percentage >30%	1 (7%)	-	1.000
Femoral fractures^1^	4 (27%)	0 (0%)	0.106
Repeat osteotomies^2 ^	1 (7%)	0	n/a

Femoral fractures within 12 months of surgery occurred in 27% of children in spica casts (four patients) compared to 0% with SLCaB (P=0.106, Table 4). All such children experienced fracture in a distant region of the femur after cast removal (non-adjacent to the initial site of surgery) and reported mechanisms included transferring (two patients) or lifting the patient (one patient), and an unknown cause (one patient).

The majority of participants in all groups (93% overall, all in the spica group (100%), and 10/12 (83%) in the SLCaB group) had their implants electively removed between six months and two years postoperatively (Figure 1). A single participant in the spica casting group had a repeat osteotomy (Figure 1, Table 4).

Complications

Nine individuals (60%) in the spica group had a complication related to immobilization compared to three (25%) SLCaB patients (P=0.248). The majority of complications were Clavien-Dindo Grade I (Table 5). For those in spica casts, peri-operative modifications were common (often ventral flaring of the abdominal portion of the cast). Despite this, pressure sores were comparable between groups at all clinic visits. Non-immobilization related complications were comparable between groups (Table 5).

**Table 5:** Clavien-Dindo classifications of complications The number of cases for each complication are denoted by n=sample size. Short leg casts and bar (SLCaB), Prescription (Rx). *Remote femoral fractures may be unrelated to immobilization type

Grade (I-V)	Immobilization-related		Non-immobilization related	
	Spica	SLCaB	Spica	SLCaB
Grade I	n=5 ventral flaring of cast; n=1 skin discoloration, observed (back)	n=1 skin discoloration, observed (thigh)	n=2 emesis, feeding intolerance; n=1 Foley re-insertion; n=1 distal limb edema, observed	n=1 emesis, feeding intolerance
Grade II	n=4 distant femur fracture, immobilized*; n=4 Rx treatment of spasm; n= 1 sacral pressure sore, topical treatment; n=1 prolonged stiffness	n=2 pharmacologic control of spasm	n=6 anemia; n=1 Rx treatment spasm; n=1 Rx treatment fluid overload; n=1 Rx treatment seizures; n=1 atelectasis; n=1 pneumonia	n=2 Rx treatment spasm; n=1 pneumonia; n=1 transfusion; n=1 Rx treatment seizures; n=1 Rx treatment gastritis, anemia
Grade IIIa	n=0	n=0	n=0	n=0
Grade IIIb	n=0	n=0	n=0	n=0
Grade IV	n=0	n=0	n=0	n=0
Grade V	n=0	n=0	n=0	n=0

## Discussion

In non-ambulatory children with quadriplegic cerebral palsy, analgesic outcomes after hip reconstruction did not significantly differ regardless of whether traditional hip spica casting or SLCaB was employed. Similarly, early orthopaedic outcomes and complication profiles appeared no different. To our knowledge, our findings related to pain and medication dosing in the peri-operative period is the first in this population. No previous report has quantified MED in children with quadriplegic cerebral palsy after hip surgery, nor attempted to quantitatively associate immobilization to opioid use. There were temporal effects in pain scores and medication administration, but no main effect of treatment (Figure 2). Thus, the null hypothesis, that those in hip spica cast would have lower pain and reduced need for opioid management, was rejected. 

Those who had SLCaB employed for postoperative immobilization did not experience any lower-extremity fractures within 12 months of hip surgery, compared to an incidence of 27% in the spica group (P=0.106). The study was not powered to detect these differences, so while not statistically significant, the complication occurred at a sufficient rate to be clinically relevant (four of 15 patients) and demands further study. This complication may be related to immobilization-mediated osteopenia, as fractures occurred distal to the surgical implants after immobilization was discontinued. There was no consistently available pre-operative bone density evaluation or activity stratification such as stander use, so the effects of daily time in a standing position or underlying metabolic bone disease is unknown.

Early radiographic outcomes of the hips at the second postoperative visit did not differ between cohorts, particularly the migration percentage, thus type of immobilization did not appear to compromise the early success of hip stabilization. Radiographic outcomes of hip reconstruction were likely a reflection of the quality and type of surgical intervention rather than the immobilization itself. 

Immobilization-related medical complications did not significantly differ between groups. Descriptively, the spica-casted group had more frequent casting adjustments in the peri-operative period; however, by the first follow-up visit those in spica casts had similar rates of pressure sores (two) compared to those in SLCaB (one). Non-immobilization related medical complications also did not differ between groups. 

Complications of spica casting have been well documented, with rates of skin complications approaching 30% in the treatment of femur fractures in developmentally normal children [12]. However, other literature is inconsistent in associating hip spica casts with complications. Lubicky et al. did not detect meaningful differences in complication rates between spica casting and “no immobilization” after spastic hip reconstruction, but Ruzbarsky et al. reviewed 93 hip reconstructions and found spica casting to be an independent risk factor for perioperative complications [11, 21]. Immediate mobilization has been reported with low complication rates after neuromuscular hip reconstruction, as has the use of an abduction pillow [20, 29]. Our complication rates are comparable to previous reports, wherein medical complications in cerebral palsy and decubitus ulcers are considered common regardless of the reason for spica cast application [10-12, 21]. 

This study has several limitations. First, the study includes demographic differences between groups (e.g., age) and hips were more subluxated in the spica group, possibly influencing postoperative pain. Second, the data were collected retrospectively; thus, the results are specific to the sample and any generalizability should be made with appropriate caution. Third, the pain scores were collected using four different pain scales. Although all scales resulted in a pain score ranging from zero to 10, the anchors guiding responses differed as do the psychometric properties of the scales. This was unavoidable as these scales were collected clinically. Finally, potential confounding variables like disuse or pharmacologic-mediated osteopenia were not available for analysis and could have influenced the rate of fragility fractures.

Despite these limitations, this is the first study of its kind to thoroughly evaluate immobilization types following hip reconstruction in children with quadriplegic cerebral palsy and the resultant impact on pain and orthopaedic outcomes. The current study suggests that there was no substantial difference in perceived pain, epidural dosage, MED administered or complication-related reason to choose one type of immobilization technique over the other. Based on this finding, future studies could be conducted prospectively in a randomized, controlled manner to further explore the impact of casting on recurrent pain during recovery, osteopenia, and determine whether individual patient characteristics could predict which immobilization technique would provide a better outcome or patient/family experience. 

## Conclusions

Pain scores, analgesic administration, and complication profiles did not differ when comparing spica casting and SLCaB immobilization following hip reconstruction in this sample of children with quadriplegic cerebral palsy. Orthopaedic success of the procedure in stabilizing the hips was not related to an immobilization regimen. In our investigation, more children treated with spica casts experienced fragility fractures of the femur, but numbers were too small to draw definitive conclusions. Prospective studies with larger sample sizes are warranted to provide clinical recommendations.
